# An MR-based radiomics model for differentiation between hepatocellular carcinoma and focal nodular hyperplasia in non-cirrhotic liver

**DOI:** 10.1186/s12957-021-02266-7

**Published:** 2021-06-21

**Authors:** Zongren Ding, Kongying Lin, Jun Fu, Qizhen Huang, Guoxu Fang, Yanyan Tang, Wuyi You, Zhaowang Lin, Zhan Lin, Xingxi Pan, Yongyi Zeng

**Affiliations:** 1 Department of Hepatopancreatobiliary Surgery, Mengchao Hepatobiliary Hospital of Fujian Medical University, Xihong Road 312, Fuzhou, 350025 China; 2 The Big Data Institute of Southeast Hepatobiliary Health Information, Mengchao Hepatobiliary Hospital of Fujian Medical University, Fuzhou, 350025 China; 3 Department of Radiology, Mengchao Hepatobiliary Hospital of Fujian Medical University, Fuzhou, 350025 China; 4 Department of Oncology, Nanhai Hospital Affiliated to Southern Medical University, Foshan, 528000 China

**Keywords:** Radiomics, Hepatocellular carcinoma, Focal nodular hyperplasia, Magnetic resonance imaging, Zongren Ding, Kongying Lin, and Jun Fu contributed equally to this work.

## Abstract

**Purpose:**

We aimed to develop and validate a radiomics model for differentiating hepatocellular carcinoma (HCC) from focal nodular hyperplasia (FNH) in non-cirrhotic livers using Gd-DTPA contrast-enhanced magnetic resonance imaging (MRI).

**Methods:**

We retrospectively enrolled 149 HCC and 75 FNH patients treated between May 2015 and May 2019 at our center. Patients were randomly allocated to a training (*n*=156) and validation set (*n*=68). In total, 2260 radiomics features were extracted from the arterial phase and portal venous phase of Gd-DTPA contrast-enhanced MRI. Using Max-Relevance and Min-Redundancy, random forest, least absolute shrinkage, and selection operator algorithm for dimensionality reduction, multivariable logistic regression was used to build the radiomics model. A clinical model and combined model were also established. The diagnostic performance of the models was compared.

**Results:**

Eight radiomics features were chosen for the radiomics model, and four clinical factors (age, sex, HbsAg, and enhancement pattern) were chosen for the clinical model. A combined model was built using the factors from the previous models. The classification accuracy of the combined model differentiated HCC from FNH in both the training and validation sets (0.956 and 0.941, respectively). The area under the receiver operating characteristic curve of the combined model was significantly better than that of the clinical model for both the training (0.984 vs. 0.937, p=0.002) and validation (0.972 vs. 0.903, p=0.032) sets.

**Conclusions:**

The combined model provided a non-invasive quantitative method for differentiating HCC from FNH in non-cirrhotic liver with high accuracy. Our model may assist clinicians in the clinical decision-making process.

## Introduction

Focal nodular hyperplasia (FNH) is the second most common benign tumor of the liver, and it is found at autopsy with a prevalence of 0.3–3% [1, 2]. It is considered a hyperplastic reaction resulting from arterial malformation, with 60–80% of cases being asymptomatic and discovered by chance [3, 4]. A typical FNH is a solitary well-defined, unencapsulated mass, with a characteristic “spoke-wheel” central scar that contains dystrophic arterial vessels on computed tomography (CT), magnetic resonance imaging (MRI), and ultrasonography (US) [5]. MRI has a higher sensitivity than US and CT and a specificity of almost 100% for the diagnosis of FNH. However, its sensitivity is lower (70–80%), especially in small FNHs where the central scar is often missing. Previous reports show 35–70% FNHs do not have this imaging feature, and atypical findings including strong hyper-intensity on T2-weighted imaging, a pseudocapsule mimicking a true capsule, and washout can result in confusion with HCC [6–8]. Due to atypical radiological features, correct diagnosis of FNH on CT and MRI may not even be possible in about 30% and 20% of cases, respectively [3, 9]. The hepatobiliary phase (HBP) of gadoxetic acid-enhanced MRI (Gd-EOBDTPA-MRI) provides valuable diagnostic information for differentiation between FNH and HCC. As 10–15% of HCCs show iso- or hyperintensity on the HBP, and approximately 73–90% of FNHs show iso- or hyperintensity on the HBP, differential diagnosis is difficult because of the overlapping features [10].

However, intractable cases must be diagnosed accurately because they require entirely different medical management. HCC is the most common primary liver cancer and the third most common cause of cancer death worldwide. Once a diagnosis of HCC has been made, intervention must be initiated. Surgical resection is a recommended treatment option in patients with resectable HCC in the absence of clinically significant portal hypertension. Other treatments including ablation, transarterial embolization and radiotherapy, transplantation, and systemic pharmacological treatment also benefit some HCC patients. Chronic hepatitis B (CHB) is the leading etiology of HCC worldwide, and most cases of HBV-related HCC (70–90%) occur in patients with cirrhosis [11, 12]. However, FNH usually occurs in livers without cirrhosis. Therefore, the need to differentiate between HCC and FNH in liver with cirrhosis background is very rare, so our study population was limited to non-cirrhotic liver. Compared with other liver lesions, the diameter of the FNH is stable in most patients and complications are extremely rare [13, 14]. The American College of Gastroenterology (ACG) Clinical Guidelines suggest that asymptomatic FNH does not require intervention [15]. Therefore, in atypical cases difficult to diagnose on imaging in a non-cirrhotic liver, biopsy is necessary [7], but it is invasive with a potential for pain and other complications [16]. Other liver lesions, such as hepatocellular adenoma (HCA), should be taken into account in the differential diagnosis. The reported prevalence of HCA is between 0.001 and 0.004%, and it is approximately 10 times less common than FNH. There are few reports on the differential diagnosis between HCA and other liver lesions. Hence, HCA was not included in our study as there were not enough cases. In summary, a non-invasive method that can distinguish HCC from FNH is desperately needed.

Radiomics using a large number of quantitative features not available to the naked eye, has been used in tumor molecular classification, differential diagnosis, treatment selection, therapeutic effect detection, and prognosis evaluation. To our best knowledge, there are few studies on the differentiation between HCC and FNH on MRI using radiomics methods. This study aimed to develop and validate a radiomics model that is non-invasive and has high accuracy for differentiating HCC from FNH in non-cirrhotic liver.

## Materials and methods

### Patients

In this single-center retrospective study, medical records were viewed to identify all consecutive cases seen between May 2015 and May 2019. The inclusion criteria applied to HCCs were (a) diagnosis with postoperative pathological evidence, (b) without radiological features of liver cirrhosis, (c) no previous history of hepatectomy or radiotherapy, and (d) HCC without blood vessels, bile duct invasion, or distant metastasis radiologically, which strongly supported the diagnosis and there was no need for it to be distinguished from benign disease. The inclusion criteria applied to FNHs were (a) postoperative pathological evidence or liver biopsy and (b) typical FNH diagnosis according to the European Association for the Study of the Liver (EASL) Clinical Practice Guidelines [7] (to improve the applicability of the model). The exclusion criteria for both HCCs and FNHs were as follows: (a) absence of high-quality pretreatment Gd-DTPA contrast-enhanced MRI (ceMRI) performed in our center, (b) MRI data obtained at least 2 months prior to the acquisition of pathological evidence, and (c) incomplete medical records and unavailability of the required clinical data. Up to three imaging studies per patient were included as long as studies were more than 6 months apart.

This study was approved by the Institutional Ethics Committee of our hospital, and written informed consent was obtained from all study participants. The studies were performed in accordance with the ethical standards outlined in the 1964 Declaration of Helsinki and its later amendments or comparable ethical standards.

### MR image acquisition and image processing

MRI examinations were performed using a 3.0 T magnetic resonance scanner (Magnetom Verio, Siemens Healthcare, Erlangen, Germany), an 8-channel phased array body coil, and a high-pressure syringe. The contrast agent was Gd-DTPA (Gd-DTPA, BeiLu Pharmaceutical Co., Ltd., Beijing, China), the dosage was 0.2 mL/kg; the speed was 2.5 mL/s, and the follow-up was rinsed with 20 mL normal saline. Preparation before the scan included fasting and no drinking for >4 h, psychological guidance, and breathing training (calm breath-holding at the end of the breath). Contrast-enhanced axial T1-weighted images (CE-T1) were acquired using a three-dimensional volumetric interpolated breath-hold examination (3D-VIBE) sequence (TR=4.16 ms, TE=2.01 ms, FOV=380×308 mm, matrix=320×320×75%, slice thickness=3 mm, spacing=3 mm, FA=16, and NEX=1) with multiphase contrast. Arterial phase (AP), portal venous phase (PVP), and delayed phase images were acquired after contrast administration at 20–30, 60–70, and 120–180 s for each patient, with breath-holding in all phases.

**Fig. 1:**
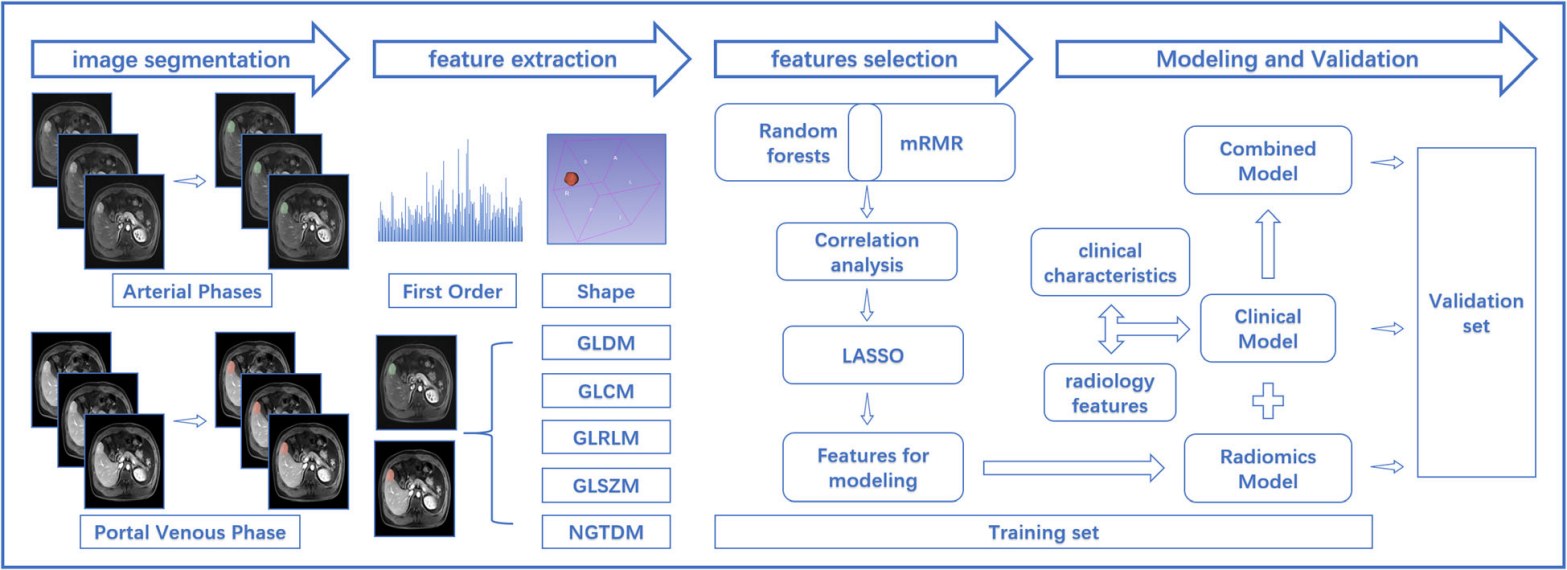
Workflow of this study. Firstly, manual segmentation was performed on arterial and portal venous phases MR image. Secondly, image preprocessing and feature extraction are carried out in the volume of interest (VOIs), including seven common feature groups: first order, shape, GLDM, GLCM, GLRLM, GLSZM, NGTDM. Thirdly, in training set, random forest algorithm and MRMR algorithm were used for pre-screening, and then, correlation analysis and LASSO regression were performed to screen out key features for modeling. Finally, three models were established: Clinical Model, Radiomics Model and Combined Model, and model performance were evaluated in validation set. Note: GLDM gray-level dependence matrix, GLCM gray-level cooccurence matrix, GLRLM gray-level run length matrix, GLSZM gray-level size zone matrix, NGTDM neigboring gray tone difference matrix, mRMR Max-Relevance and Min-Redundancy, LASSO the least absolute shrinkage and selection operator algorithm

The FNH and HCC lesions were segmented manually using a 3D-Slicer (version 4.10.2; http://www.slicer.org). The AP and PVP of T1 images were used to indicate the volumes of interest (VOIs) by drawing the outline of tumor tissue layer-after-layer and avoiding the bile duct and vessels by Radiologists 1 and 2. If there were multiple lesions, only the largest lesions were segmented. PyRadiomics (version 2.1; http://www.radiomics.io/) implementation in 3D-Slicer was utilized for further preprocessing and radiomics feature extraction. We adopted resampling as a preprocessing method, which was performed to obtain a voxel size of 1×1×1 mm^3^ via trilinear interpolation before feature calculation [17]. A fixed bin width of 25 was used for the image discretization. Image reconstruction was performed by applying wavelet decomposition filtering and Laplacian of Gaussian filtering with sigma values of 0.5, 1.0, and 1.5. Seven common feature groups were extracted from filtered and original images in three dimensions, including a first order, gray-level dependence matrix (GLDM), gray-level co-occurrence matrix (GLCM), gray-level run length matrix (GLRLM), gray-level size zone matrix (GLSZM), neighboring gray tone difference matrix (NGTDM), and shape (Fig. 1).

### Inter-observer and intra-observer agreement

Inter- and intra-observer correlation coefficients (ICC) were used to evaluate the inter-observer reliability and intra-observer reproducibility of feature extraction [18]. Thirty samples were randomly chosen and delineated by two radiologists. Radiologist 1 delineated the VOIs on AP and PVP of T1 images twice within 1 week under the same standard to assess intra-observer reproducibility, and Radiologist 2 independently delineated the VOIs once to assess inter-observer agreement by comparing the results with the radiomics features extracted from the VOIs delineated by Radiologist 1 [18]. Radiomics features were selected when the ICC was >0.8. Radiologist 1 finished the remaining samples.

### Radiomics feature selection and model construction

Before radiomics feature selection, z score normalization was employed to eliminate different feature magnitudes by scaling values to a mean of 0 and a standard deviation of 1 [18]. Then, the samples were randomly grouped into training (n=156) and validation sets (n=68). The training set was used for radiomics feature selection and construction of the three models. The validation set was used to evaluate the diagnostic performance of the three models. We used two machine learning algorithms: (i) Max-Relevance and Min-Redundancy (mRMR) and (ii) random forests (RF). Each algorithm selects the top 20 features with the highest score or the highest importance features. A correlation analysis was carried out to exclude the features with high correlation. Least absolute shrinkage and selection operator (LASSO) regression was employed for the next step selection of features, with penalty parameter tuning conducted by 10-fold cross-validation to compile a radiomics signature [18–24]. The optimal radiomics signature was used to create the radiomics model.

### Construction of the clinical model and combined model

Univariate analysis was applied to compare the differences in clinical factors (including clinical information and MR features) between the two groups, and a multiple logistic regression analysis was used to build the clinical model using the significant variables from the univariate analysis as inputs. Odds ratios (ORs) as estimates of relative risk with 95% confidence intervals (CI) were obtained for each risk factor. The combined model was built using the clinical factors in the clinical model and Rad score in the radiomics model.

### Statistics

Statistical analysis was performed using R (version 3.6.3; R Foundation for Statistical Computing, Vienna, Austria). Categorical variables were compared using the χ^2^ test or Fisher’s exact test. Continuous variables were expressed as the median [Q1, Q3] and compared using the Student’s t test or Mann-Whitney U test. Variables that reached statistical significance in the univariate analysis were considered for the multivariate binary logistic regression model. mRMR, RF, and LASSO were implemented using “mRMRe,” “randomForest,” and “glmnt,” respectively. The Delong test was used to measure the differences in the ROC curves [25]. P<0.05 was considered statistically significant.

## Results

### Patient characteristics

**Fig. 2:**
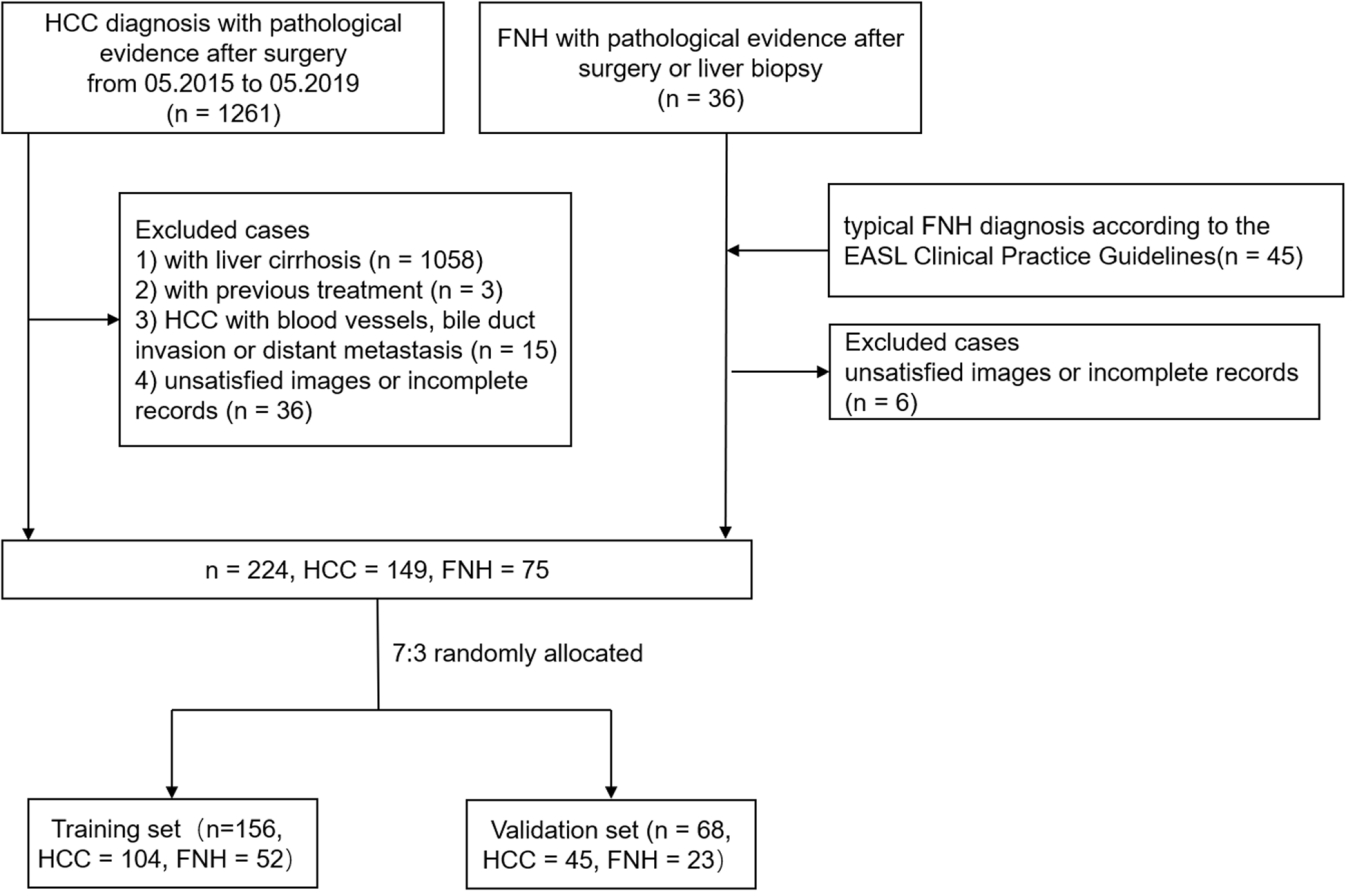
Flow chart of patient recruitment in this study. Note: HCC hepatocellular carcinoma in noncirrhotic liver, FNH focal nodular hyperplasia

Our study selection process is described in Fig. 2. The cases search in our medical records generated 1261 HCC and 36 FNH with pathological evidence between May 2015 and May 2019. Of those HCC candidates, 1058 cases with liver cirrhosis; 3 cases with previous treatment; 15 cases with blood vessels, bile duct invasion or distant metastasis; and 36 cases with unsatisfied images or incomplete records were excluded. Based on 36 cases of FNH, 45 typical FNH diagnosed according to the EASL Clinical Practice Guidelines were added, and 6 cases were excluded due to unsatisfied images or incomplete records.

Finally, a total of 224 patients with HCC (n=149, 124 men and 25 women; mean age, 56.8±11.9 years) and FNH (n=75, 30 men and 45 women; mean age, 37.0±12.1 years) were enrolled. The clinical factors of FNHs and HCCs in the training and validation sets are shown in Table 1. All clinical factors showed no significant difference between sets (P>0.05).

**Table 1:** Clinical factors of the training and validation sets

Clinical factors	Training set (***n*** = 156)		***P***	Validation set (***n*** = 68)			
	HCC (***n*** = 104)	FNH (***n*** = 52)		HCC (***n*** = 45)	FNH (***n*** = 23)	***P***	P*
**Gender,** male/female	84/20	18/34	0.001	40/5	12/11	0.002	0.136
**Age,** ≤50/>50 years	33/71	45/7	0.001	12/33	19/4	0.001	0.644
**Hbsag,** negative/positive	21/83	38/14	0.001	16/29	16/7	0.016	0.252
**AFP,** ≤400/> 400 ng/ml	99/5	52/0	0.261	45/0	23/0	-	0.317
**MRI tumour number,** single/multiple	89/15	36/16	0.028	40/5	16/7	0.101	0.838
**MRI tumour size,** cm	4.35 [2.60, 6.25]	2.50 [1.90, 3.33]	0.001	4.40 [3.00, 6.20]	2.70 [2.05, 3.95]	0.006	0.849
**Liver steatosis,** absent/present	78/26	39/13	1.000	33/12	20/3	0.331	0.762
**Liver haemangioma,** absent/present	88/16	49/3	0.141	42/3	22/1	1.000	0.235
**Location,** internal/subcapsular	36/68	30/22	0.010	16/29	11/12	0.474	0.829
**Margin,** ill-defined/well-defined	26/78	11/41	0.739	11/34	3/20	0.434	0.734
**Shape,** not round/round	46/58	29/23	0.234	18/27	16/7	0.040	0.905
**Pseudocapsule,** absent/present	67/37	28/24	0.270	24/21	13/10	1.000	0.448
**Lesion homogeneity,** Heterogeneous/homogeneous	68/36	21/31	0.005	29/16	7/16	0.016	0.672
**Lesion with steatosis,** absent/present	86/18	49/3	0.082	38/7	22/1	0.337	0.895
**Central vascular supply,** absent/present	90/14	49/3	0.238	36/9	21/2	0.396	0.380
**Central scar,** absent/present	82/22	37/15	0.387	40/5	14/9	0.017	0.734
**Enhancement pattern**			0.001			0.001	0.56
Early enhancement + washout	82	15	-	36	7	-	-
Early enhancement + no washout	14	35	-	7	16	-	-
Other patterns	8	2	-	2	0	-	-

Note: *HCC* hepatocellular carcinoma in noncirrhotic liver, *FNH* focal nodular hyperplasia, *HbsAg* hepatitis B surface antigen, *AFP* alpha fetoprotein; P* represents the *P* value of comparison between training and validation set

### Inter- and intra- observer agreement

We extracted 2260 radiomics features in each patient from the AP and PVP of the ceMRI. Intra-observer agreement was 85.3% (mean ICC=0.90). Inter-observer agreement for all 2260 features reached 78.4% (mean ICC=0.85). Seven hundred and thirty-one features were excluded.

### Radiomics feature selection and model construction

**Fig. 3:**
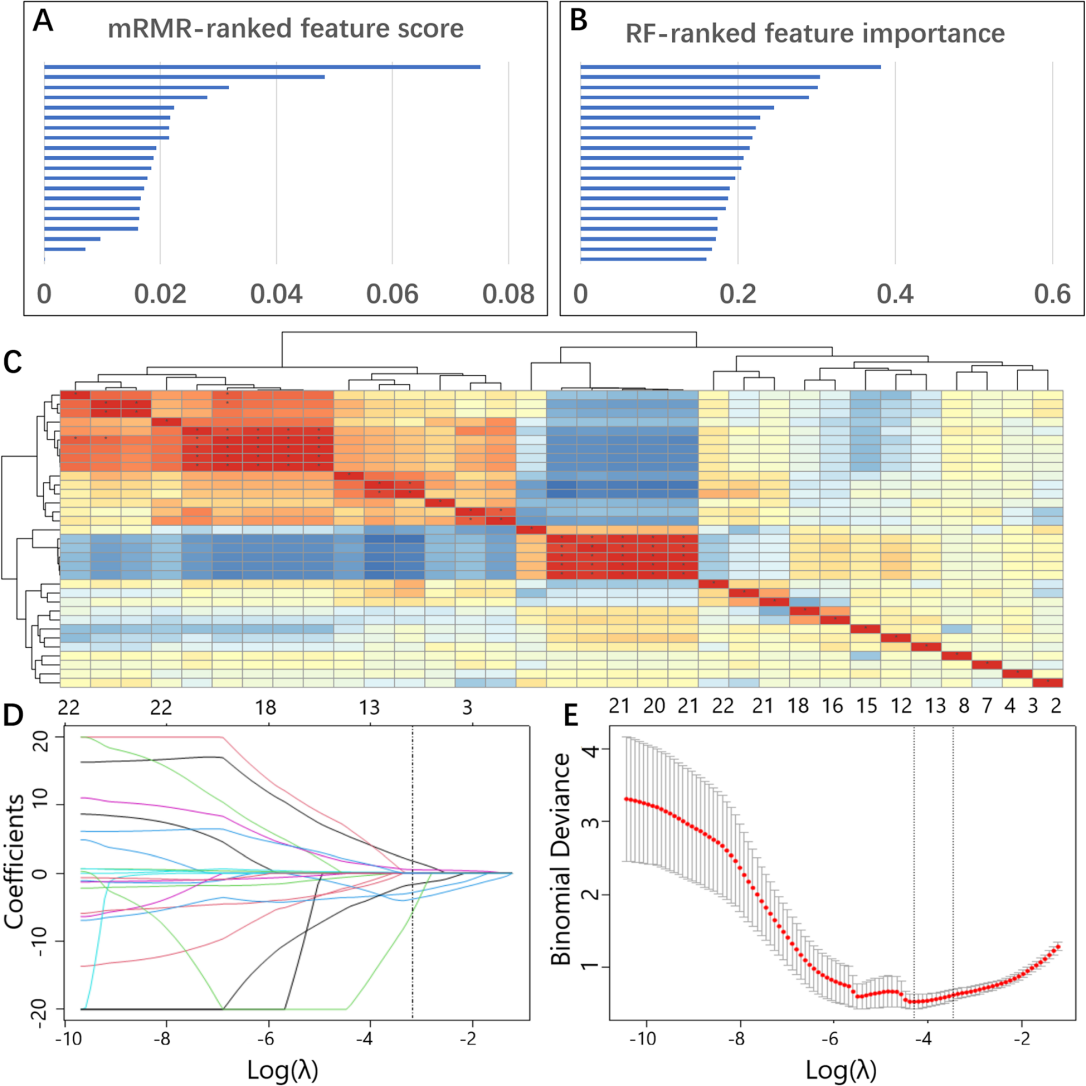
Dimensionality reduction and Radiomics Model construction. **a** The 20 features selected by the mRMR algorithm according to features score. **b** The 20 features selected by the Random forests algorithm according to features importance. **c** The correlation analysis heatmap of 33 features screened by the two algorithms above (seven overlapping features were removed). **d** LASSO regression analysis of 33 features, the vertical line shows the optimal value of λ= 0.041 and 8 corresponding features with non-zero coefficients. **e** The AUC curve was plotted by tuning parameter (λ) selection performed by 10-fold cross-validation. Vertical lines on the left and right denote the minimum criterion and 1-standard error criterion (1-SE), respectively. The 1-SE criterion was applied

**Fig. 4:**
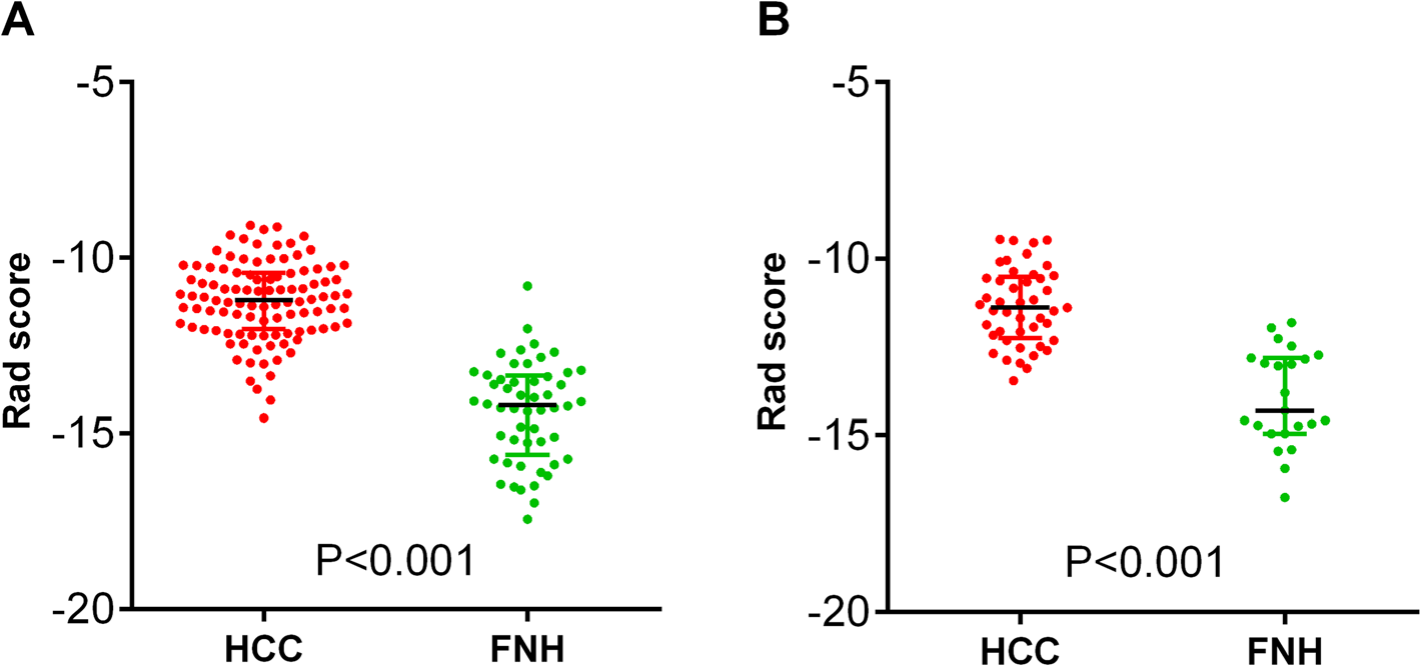
Rad score of NC-HCC and FNH in training (**a**) and validation (**b**) set. NC-HCC has a higher Rad score than FNH both in training and validation set. Rad score=-6.68*(PVP-glcm-wavelet-HHL-InverseVariance)-3.87*(AP-firstorder-original-10Percentile)-2.81*(PVP-glcm-log-sigma-1-5-mm-3D-MaximumProbability)-1.65*(PVP-glcm-MaximumProbability)+0.08*(AP-glcm-log-sigma-1-0-mm-3D-ClusterShade)+0.11*(PVP-firstorder-wavelet-HLL-Median)+0.54*(AP-firstorder-log-sigma-0-5-mm-3D-Median)+1.81*(AP-shape-original-Elongation)

After combining the top 20 engineered features ranked by the mRMR and RF algorithms, 33 features were identified from the training set, with seven features selected simultaneously by two algorithms (Fig. 3). Pearson correlation analysis of the 33 features showed that 11 pairs of features were highly correlated (coefficients >0.80). Twenty-two features were subjected to LASSO regression, and eight features were selected with the best tuned regularization parameter λ of 0.041 under the 1-SE criterion found by 10-fold cross validation. The selected features were calculated according to the following formula to build a radiomics model: Rad score = −6.68 * (PVP-glcm-wavelet-HHL-InverseVariance) – 3.87 * (AP-firstorder-original-10Percentile) – 2.81 * (PVP-glcm-log-sigma-1-5-mm-3D-MaximumProbability) – 1.65 * (PVP-glcm-MaximumProbability) + 0.08 * (AP-glcm-log-sigma-1-0-mm-3D-ClusterShade) + 0.11 * (PVP-first order-wavelet-HLL-Median) + 0.54 * (AP-firstorder-log-sigma-0-5-mm-3D-Median) + 1.81 * (AP-shape-original-Elongation). The Mann-Whitney U test of the Rad score was performed in the training and validation sets, and statistically significant differences were found between the two sets (Fig. 4).

### Construction of the clinical model and combined model

Univariate analysis showed that age, sex, HbsAg, MRI tumor size, MRI tumor number, location, lesion homogeneity, and enhancement pattern reached statistical significance (P<0.05). Multivariate analysis showed that age (OR=11.09 [3.13–49.40], P<0.001), sex (OR=5.57 [1.74–19.85], P=0.005), HbsAg (OR=14.75 [4.43–60.94], P<0.001), and enhancement pattern (OR=0.21 [0.07–0.52], P=0.001) were independent predictors for differential diagnosis of HCC and FNH, and they were used to build the clinical model. A combined model was also built using the four clinical factors and Rad score by logistic regression.

### Diagnostic performance of the three models

Good performance of the clinical model, radiomics model, and combined model for the training set was observed, with an area under the curve (AUC) of 0.937 (95% CI 0.887–0.970), 0.960 (95% CI 0.916–0.985), and 0.984 (95% CI 0.949–0.997), with a classification accuracy of 0.853, 0.917, and 0.956, respectively. When comparing the AUCs between the three models, the combined model proved to be significantly better than the clinical model (P=0.002), but the difference between the clinical model and radiomics model was not statistically significant.

Consistent results were obtained in the validation set. The AUC of the clinical model, radiomics model, and combined model for the validation set was 0.903 (95% CI 0.807–0.962), 0.931 (95% CI 0.843–0.978), and 0.972 (95% CI 0.900–0.997), with a classification accuracy of 0.853, 0.868, and 0.941, respectively. When comparing the AUCs between the three models, the combined model proved to be significantly better than the clinical model (P=0.032), but the difference between the clinical model and radiomics model was not statistically significant (Table 2).

**Table 2:** Model performance in the training and validation sets

Model	Cutoff	AUC(95%CI)	Specificity	Sensitivity	Accuracy	***P***
**Training set**						
Clinical Model	0.684	0.937(0.887-0.970)	0.923	0.817	0.853	Ref
Radiomics Model	0.695	0.960(0.916-0.985)	0.942	0.904	0.917	0.252
Combined Model	0.607	0.984(0.949-0.997)	0.962	0.952	0.956	0.002
**Validation set**						
Clinical Model	0.625	0.903(0.807-0.962)	0.826	0.867	0.853	Ref
Radiomics Model	0.658	0.931(0.843-0.978)	0.826	0.889	0.868	0.535
Combined Model	0.859	0.972(0.900-0.997)	0.957	0.933	0.941	0.032

Note: *AUC* area under the curve, *CI* confidence interval

## Discussion

**Fig. 5:**
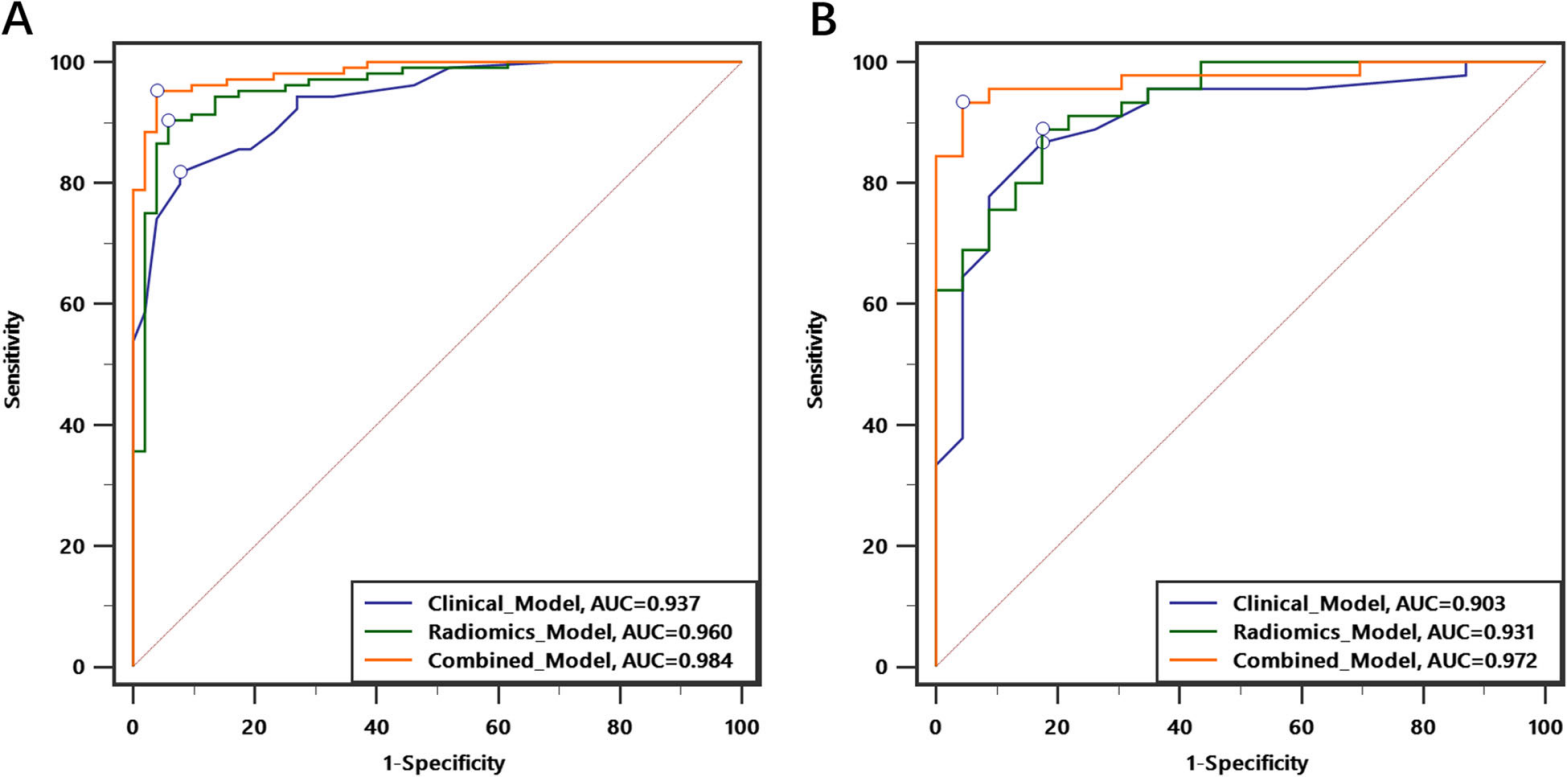
ROC curves comparing the three models in training (**a**) and validation (**b**) set. The hollow point represents the optimal cut-off value on the curve

In this study, we established three models to distinguish HCC from FNH in non-cirrhotic liver using four clinical factors and a Rad score, which was combined with eight radiomics features filtrated from AP and PVP on MRI. In comparison to the clinical model, the combined model showed overall superiority in the evaluation of accuracy, sensitivity, specificity, and AUC in both the training and validation sets (Table 2, Fig. 5). The addition of radiomics features improved the performance of the diagnostic model, but the radiomics model did not bring significant improvement compared to the clinical model.

Many previous studies have provided several ways to differentiate HCC from FNH. Li et al. [26] enrolled 38 patients with HCC and 65 with FNH to assess the diagnostic ability of contrast-enhanced US (ceUS) and microflow (MF) imaging and found that MF imaging had an excellent diagnostic performance in terms of differentiating between atypical HCC and FNH compared to routine ceUS. Yu et al. [27] included 42 HCCs and 16 FNHs and performed spectral CT during the arterial and portal venous phases and found that CT spectral imaging increased the detectability and accuracy of differentiation between HCC and FNH. Nie et al. [28] developed and validated a CT-based radiomics nomogram for preoperative differentiation of FNH from HCC in livers without cirrhosis, achieving an AUC of 0.917 in the validation group. Several studies [29–31] have indicated that Gd-EOBDTPA-MRI is helpful for the diagnosis of FNH, as most FNHs show high- or iso-signal intensity (SI) compared to liver parenchyma in the hepatobiliary phase (HBP). However, Lee et al. [32] found that 85% of well-differentiated HCCs were hypointense on HBP, and about 15% of well-differentiated HCCs were iso- or hyperintense on HBP, illustrating that there is still some overlap between them, even in the HBP. In this study, we established a combined model for differential diagnosis of HCC from FNH in non-cirrhotic livers. Our model is non-invasive and easy to implement, and it achieved excellent performance with an AUC of 0.972 in the validation set.

In our study, the clinical model did not achieve the best AUC (0.937 and 0.903 in the training and validation sets, respectively), but it was still relatively high. Although we included as many of the radiological features that we could to help identify the two diseases as mentioned in the EASL Clinical Practice Guide for benign liver tumors, such as liver hemangioma, steatosis in lesions, and the liver, they turned out not to be strong predictors. The presence of a central scar is a typical feature of FNH, which is identified on MRI in approximately 30–50% of FNH cases [8]. On the other hand, about 50% of non-cirrhotic HCCs have a central scar detectable by MRI, especially in fibrolamellar carcinoma [33]. In our study, 32% of FNHs had a central scar, which was consistent with previous studies. In our study, the central scar was ultimately not included in the model.

Our results were consistent with the study reported by Nie et al. [28]. They also included only one radiological feature in their model, which was enhancement pattern, as we did. The epidemiological and clinical characteristics of these two diseases are also important references for differential diagnosis. FNH mainly occurs in females (up to 90% of cases), with an average age between 35 and 50 years. HCC mainly occurs in elderly males, usually accompanied by hepatitis B virus infection. Clinical factors—age, sex, and HbsAg—were consistent with the epidemiological differences between the two diseases, indicating the interpretability of our models.

Radiomics includes an enormous amount of data with high-dimensional characteristics, so it is important to know how to extract the key features from such a huge amount of data. In order to ensure the reproducibility of the selected features and avoid the interference by other subjective factors, we implemented rigorous feature selection in combination with machine learning. First, inter-observer and intra-observer agreements were evaluated, and features with an ICC >0.8 were included. Second, two machine learning algorithms, mRMR and RF, were used for feature filtering. Third, a correlation analysis of the features screened by the two algorithms was performed to exclude features of high correlation. Finally, LASSO regression, one of the most commonly used methods for dimensionality reduction in radiomics, was used to obtain the optimal radiomics signature.

There are some differences between our model and existing diagnostic techniques. The existing diagnostic techniques mainly use enhanced CT, enhanced MR, or contrast-enhanced ultrasound examination to observe the imaging findings of the lesions. Meanwhile, the baseline data of the patients, such as gender, age, AFP, and background of cirrhosis, are also important references. LI-RADS standard was used as the diagnostic criteria for HCC, and EASL Clinical Practice Guidelines was used for FNH diagnosis [7, 34]. Since imaging diagnosis depends on subjective judgment, not all HCC or FNH have typical imaging findings, and heterogeneity between observers is strong, atypical cases can only be confirmed by invasive pathological evidence (surgery or biopsy). Unlike existing diagnostic technology, we established “An MR-based radiomics model” based on radiomics features extracted form MR images, combined with patient baseline characteristics, made diagnosis using mathematical model based on the objective parameters, and achieved an AUC of 0.984 and 0.972 in the training and validation sets. Furthermore, our model is the first study to use MR radiomics model for the differential diagnosis of HCC and FNH.

Our study had several limitations. First, the number of samples was still limited compared to the large number of features. A large-scale clinical study enrolling more samples would help validate and improve the applicability of our model as an effective tool for differentiating between FNH and HCC. Second, external validation is needed to further verify the accuracy and clinical practicability of the model. Finally, sample selection bias was unavoidable in this retrospective study. Therefore, a prospective study should be conducted to further prove the practicability of the model.

In conclusion, our novel MR-based radiomics model demonstrated a powerful diagnostic capability because of its excellent performance, with a certain reference value for differentiating HCC from FNH in clinical studies.

## Data Availability

The datasets generated and/or analyzed during the current study are not publicly available due [We plan to use this data set to publish another paper in a different research area] but are available from the corresponding author on reasonable request.

## References

[1] Lewis RB, Lattin GE, Makhlouf HR, Levy AD (2010). Tumors of the liver and intrahepatic bile ducts: radiologic-pathologic correlation. Magn Reson Imaging Clin N Am.

[2] Karhunen PJ (1986). Benign hepatic tumours and tumour like conditions in men. J Clin Pathol.

[3] Cherqui D, Rahmouni A, Charlotte F, Boulahdour H, Metreau JM, Meignan M, Fagniez PL, Zafrani ES, Mathieu D, Dhumeaux D (1995). Management of focal nodular hyperplasia and hepatocellular adenoma in young women: a series of 41 patients with clinical, radiological, and pathological correlations. Hepatology.

[4] Luciani A, Kobeiter H, Maison P, Cherqui D, Zafrani ES, Dhumeaux D, Mathieu D (2002). Focal nodular hyperplasia of the liver in men: is presentation the same in men and women?. Gut.

[5] Giambelluca D, Taibbi A, Midiri M, Bartolotta TV (2019). The “spoke wheel” sign in hepatic focal nodular hyperplasia. Abdom Radiol (NY).

[6] Kamel IR, Liapi E, Fishman EK (2006). Focal nodular hyperplasia: lesion evaluation using 16-MDCT and 3D CT angiography. AJR Am J Roentgenol.

[7] EASL (2016). EASL Clinical Practice Guidelines on the management of benign liver tumours. J Hepatol.

[8] Murakami T, Tsurusaki M (2014). Hypervascular benign and malignant liver tumors that require differentiation from hepatocellular carcinoma: key points of imaging diagnosis. Liver Cancer.

[9] Choi CS, Freeny PC (1998). Triphasic helical CT of hepatic focal nodular hyperplasia: incidence of atypical findings. AJR Am J Roentgenol.

[10] Suh CH, Kim KW, Kim GY, Shin YM, Kim PN, Park SH (2015). The diagnostic value of Gd-EOB-DTPA-MRI for the diagnosis of focal nodular hyperplasia: a systematic review and meta-analysis. Eur Radiol.

[11] Kulik L, El-Serag HB (2019). Epidemiology and management of hepatocellular carcinoma. Gastroenterology.

[12] Bruix J, Reig M, Sherman M (2016). Evidence-based diagnosis, staging, and treatment of patients with hepatocellular carcinoma. Gastroenterology.

[13] D'Halluin V, Vilgrain V, Pelletier G, Rocher L, Belghiti J, Erlinger S, Buffet C (2001). Natural history of focal nodular hyperplasia. A retrospective study of 44 cases. Gastroenterol Clin Biol.

[14] Perrakis A, Demir R, Muller V, Mulsow J, Aydin U, Alibek S, Hohenberger W, Yedibela S (2012). Management of the focal nodular hyperplasia of the liver: evaluation of the surgical treatment comparing with observation only. Am J Surg.

[15] Marrero JA, Ahn J, Rajender Reddy K (2014). G. Americal College of, ACG clinical guideline: the diagnosis and management of focal liver lesions. Am J Gastroenterol.

[16] Bravo AA, Sheth SG, Chopra S (2001). Liver biopsy. N Engl J Med.

[17] Ji GW, Zhu FP, Xu Q, Wang K, Wu MY, Tang WW, Li XC, Wang XH (2019). Machine-learning analysis of contrast-enhanced CT radiomics predicts recurrence of hepatocellular carcinoma after resection: a multi-institutional study. EBioMedicine.

[18] Chen Y, Chen TW, Wu CQ, Lin Q, Hu R, Xie CL, Zuo HD, Wu JL, Mu QW, Fu QS, Yang GQ, Zhang XM (2019). Radiomics model of contrast-enhanced computed tomography for predicting the recurrence of acute pancreatitis. Eur Radiol.

[19] Fan Y, Chen C, Zhao F, Tian Z, Wang J, Ma X, Xu J (2019). Radiomics-based machine learning technology enables better differentiation between glioblastoma and anaplastic oligodendroglioma. Front Oncol.

[20] Liu F, Ning Z, Liu Y, Liu D, Tian J, Luo H, An W, Huang Y, Zou J, Liu C, Liu C, Wang L, Liu Z, Qi R, Zuo C, Zhang Q, Wang J, Zhao D, Duan Y, Peng B, Qi X, Zhang Y, Yang Y, Hou J, Dong J, Li Z, Ding H, Zhang Y, Qi X (2018). Development and validation of a radiomics signature for clinically significant portal hypertension in cirrhosis (CHESS1701): a prospective multicenter study. EBioMedicine.

[21] Kim JY, Park JE, Jo Y, Shim WH, Nam SJ, Kim JH, Yoo R-E, Choi SH, Kim HS (2019). Incorporating diffusion- and perfusion-weighted MRI into a radiomics model improves diagnostic performance for pseudoprogression in glioblastoma patients. Neuro-oncology.

[22] Zhang Y, Zhu Y, Zhang K, Liu Y, Cui J, Tao J, Wang Y, Wang S (2020). Invasive ductal breast cancer: preoperative predict Ki-67 index based on radiomics of ADC maps. Radiol Med.

[23] Hu J, Wang T, Zhang K-H, Jiang Y-P, Xu S, Chen S-H, He Y-T, Yuan H-L, Wang Y-Q (2019). Pretreatment risk management of a novel nomogram model for prediction of thoracoabdominal extrahepatic metastasis in primary hepatic carcinoma. J Transl Med.

[24] Huang X, Long L, Wei J, Li Y, Xia Y, Zuo P, Chai X (2019). Radiomics for diagnosis of dual-phenotype hepatocellular carcinoma using Gd-EOB-DTPA-enhanced MRI and patient prognosis. J Cancer Res Clin Oncol.

[25] DeLong ER, DeLong DM, Clarke-Pearson DL (1988). Comparing the areas under two or more correlated receiver operating characteristic curves: a nonparametric approach. Biometrics.

[26] Li W, Wang W, Liu GJ, Chen LD, Wang Z, Huang Y, Liu JY, Xie XY, Lu MD (2015). Differentiation of atypical hepatocellular carcinoma from focal nodular hyperplasia: diagnostic performance of contrast-enhanced US and microflow imaging. Radiology.

[27] Yu Y, Lin X, Chen K, Chai W, Hu S, Tang R, Zhang J, Cao L, Yan F (2013). Hepatocellular carcinoma and focal nodular hyperplasia of the liver: differentiation with CT spectral imaging. Eur Radiol.

[28] Nie P, Yang G, Guo J, Chen J, Li X, Ji Q, Wu J, Cui J, Xu W (2020). A CT-based radiomics nomogram for differentiation of focal nodular hyperplasia from hepatocellular carcinoma in the non-cirrhotic liver. Cancer Imaging.

[29] An HS, Park HS, Kim YJ, Jung SI, Jeon HJ (2013). Focal nodular hyperplasia: characterisation at gadoxetic acid-enhanced MRI and diffusion-weighted MRI. Br J Radiol.

[30] Bieze M, van den Esschert JW, Nio CY, Verheij J, Reitsma JB, Terpstra V, van Gulik TM, Phoa SSKS (2012). Diagnostic accuracy of MRI in differentiating hepatocellular adenoma from focal nodular hyperplasia: prospective study of the additional value of gadoxetate disodium. AJR Am J Roentgenol.

[31] Grazioli L, Bondioni MP, Haradome H, Motosugi U, Tinti R, Frittoli B, Gambarini S, Donato F, Colagrande S (2012). Hepatocellular adenoma and focal nodular hyperplasia: value of gadoxetic acid-enhanced MR imaging in differential diagnosis. Radiology.

[32] Lee MH, Kim SH, Park MJ, Park CK, Rhim H (2011). Gadoxetic acid-enhanced hepatobiliary phase MRI and high-b-value diffusion-weighted imaging to distinguish well-differentiated hepatocellular carcinomas from benign nodules in patients with chronic liver disease. AJR Am J Roentgenol.

[33] Desai A, Sandhu S, Lai JP, Sandhu DS (2019). Hepatocellular carcinoma in non-cirrhotic liver: a comprehensive review. World J Hepatol.

[34] Chernyak V, Fowler KJ, Kamaya A, Kielar AZ, Elsayes KM, Bashir MR, Kono Y, Do RK, Mitchell DG, Singal AG, Tang A, Sirlin CB (2018). Liver Imaging Reporting and Data System (LI-RADS) Version 2018: Imaging of hepatocellular carcinoma in at-risk patients. Radiology.

